# Continuously sutured versus linear-stapled anastomosis in robot-assisted hybrid Ivor Lewis esophageal surgery following neoadjuvant chemoradiotherapy: a single-center cohort study

**DOI:** 10.1007/s00464-022-09415-3

**Published:** 2022-07-19

**Authors:** Fiorenzo V. Angehrn, Kerstin J. Neuschütz, Lana Fourie, Pauline Becker, Markus von Flüe, Daniel C. Steinemann, Martin Bolli

**Affiliations:** 1 Department of Surgery, Clarunis AG – University Center for Gastrointestinal and Liver Diseases, Postfach, 4002 Basel, Switzerland; 2 Department of Surgery, University Hospital Basel, Spitalstrasse 23, 4031 Basel, Switzerland

**Keywords:** Robotic surgery, Esophageal cancer, Neoadjuvant therapy, Gastroesophageal anastomosis, Continuous suture, Stapled suture

## Abstract

**Background:**

Esophageal cancer surgery is technically highly demanding. During the past decade robot-assisted surgery has successfully been introduced in esophageal cancer treatment. Various techniques are being evaluated in different centers. In particular, advantages and disadvantages of continuously sutured (COSU) or linear-stapled (LIST) gastroesophageal anastomoses are debated. Here, we comparatively analyzed perioperative morbidities and short-term outcomes in patients undergoing hybrid robot-assisted esophageal surgery following neoadjuvant chemoradiotherapy (nCRT), with COSU or LIST anastomoses in a single center.

**Methods:**

Following standardized, effective, nCRT, 53 patients underwent a hybrid Ivor Lewis robot-assisted esophagectomy with COSU (*n* = 32) or LIST (*n* = 21) gastroesophageal anastomoses. Study endpoints were intra- and postoperative complications, in-hospital morbidity and mortality. Duration of operation, intensive care unit (ICU) and overall hospital stay were also evaluated. Furthermore, rates of rehospitalization, endoscopies, anastomotic stenosis and recurrence were assessed in a 90-day follow-up.

**Results:**

Demographics, ASA scores and tumor characteristics were comparable in the two groups. Median duration of operation was similar in patients with COSU and LIST anastomosis (467 vs. 453 min, IQR 420–521 vs. 416–469, *p* = 0.0611). Major complications were observed in 4/32 (12.5%) and 4/21 (19%) patients with COSU or LIST anastomosis, respectively (*p* = 0.697). Anastomotic leakage was observed in 3/32 (9.3%) and 2/21 (9.5%) (*p* = 1.0) patients with COSU or LIST anastomosis, respectively. Pleural empyema occurred in 1/32 (3.1%) and 2/21 (9.5%) (*p* = 0.555) patients, respectively. Mortality was similar in the two groups (1/32, 3.1% and 1/21, 4.7%, *p* = 1.0). Median ICU stay did not differ in patients with COSU or LIST anastomosis (*p* = 0.255), whereas a slightly, but significantly (*p* = 0.0393) shorter overall hospital stay was observed for COSU, as compared to LIST cohort (median: 20 vs. 21 days, IQR 17–22 vs. 18–28).

**Conclusions:**

COSU is not inferior to LIST in the performance of gastroesophageal anastomosis in hybrid Ivor Lewis operations following nCRT.

**Supplementary Information:**

The online version contains supplementary material available at 10.1007/s00464-022-09415-3.

The incidence of esophageal cancer is decreasing in Europe, but this malignancy is still diagnosed in about 5/100.000 men and 1/100.000 women each year [1]. Tumor excision is technically highly demanding [2, 3], and open surgery is increasingly being replaced by less invasive procedures [4]. Moreover, in the past decade, the improved surgical accuracy of robot-assisted esophagectomy, due to enhanced three-dimensional view and freedom of motion, has repeatedly been documented [4–9]. However, techniques of robot-assisted esophagectomy are far from standardized and a variety of aspects are still being actively investigated.

Stapling has been shown to facilitate and accelerate the creation of the anastomosis in different surgeries [10]. However, stapling might also prove to be technically challenging and associated complications have been reported [11, 12].

Previous studies have suggested a superiority of stapled in comparison to continuously sewn gastroesophageal anastomoses in open and minimally invasive Ivor Lewis operations [5, 7, 13–15].

A recent meta-analysis regarding robot-assisted esophagectomy has examined a number of reports using circular (CIST) or linear stapling (LIST), or continuous suture (COSU) to the perform gastroesophageal anastomosis [16]. CIST is frequently used during robot-assisted approaches. However, maneuvering of the rigid stapler in the narrow intercostal space, limited by the multiple robotic arms, is challenging and the undocking of the robotic arms and performance of this step thoracoscopically has been recommended [16]. Although the creation of COSU and LIST anastomoses is technically more demanding compared to CIST, these techniques are frequently preferred since they require less bedside support [16]. However, there are no comparative studies on COSU and LIST anastomoses in robot-assisted Ivor Lewis operations performed by the same surgical team.

Importantly, neoadjuvant chemoradiotherapy (nCRT), is currently emerging as standard treatment prior to esophagectomy [17–20]. However, nCRT has been associated with increased complication rates and postoperative mortality [21, 22]. Moreover, the impact on clinical outcome of defined steps of nCRT, such as, for instance, its timing before surgery, is still debated [23, 24]. Most remarkably, previous studies on gastroesophageal anastomosis techniques only included variable percentages of nCRT-treated patients, and the use of standardized treatment protocols was not considered [16].

To fill these knowledge gaps, in this study we comparatively analyzed perioperative morbidity and surgical outcomes in patients treated with a highly standardized and effective nCRT protocol, undergoing a hybrid robot-assisted Ivor Lewis esophagectomy with LIST or COSU gastroesophageal anastomosis in a single center.

## Patients and methods

### Patients

The study included all patients with esophageal cancer treated with nCRT (see below) prior to undergoing robot-assisted surgery at the University Center for Gastrointestinal and Liver Disease (Clarunis, Basel, Switzerland) between 2015 and 2020. Exclusion criteria were emergency surgery, missing nCRT, other surgical procedures, and primary fully open surgery. In our institution circular stapled esophagogastrostomies are not performed during robot-assisted Ivor Lewis procedures. Thus, we have no experience to report about this anastomotic technique and will therefore not address it within this study.

The study was approved by the Ethical Committee of Northwestern Switzerland.

### Data collection

Patients’ data were prospectively recorded in an institutional study registry data base. They included demographic data, UICC TNM staging prior to and after nCRT, American Society of Anesthesiology (ASA) classification, anastomotic technique, operation time, recorded as time from skin incision to wound closure, histopathological evaluation, morbidity (see below), mortality, and postoperative duration of hospitalization, including length of stay in the intensive care unit (ICU).

### Study endpoints

Primary study endpoints were represented by intra- and postoperative complications and in-hospital mortality. Complications were defined as any type of event requiring a deviation during surgery or within the postoperative course, and their severity was assessed using the Clavien–Dindo classification [25]. In particular, minor morbidity was defined as a Clavien–Dindo score of IIIa or below and major morbidity as IIIb or above. The Comprehensive Complication Index [26] was also assessed. Furthermore, the duration of operation, intensive care unit (ICU) and overall hospital stay were evaluated. Additionally, the rate of rehospitalization, performed endoscopies, anastomotic stenoses as well as the rate of recurrence and mortality were assessed within a 90-day follow-up.

### Statistical analysis

Continuous data were evaluated by calculating mean and standard deviation (SD) values for normally distributed data, or medians and interquartile ranges (IQR) for non-normally distributed data. Significance of observed differences was evaluated by Student’s t-test and Mann–Whitney U test, as appropriate. Categorical data were reported as counts and/or percentages and they were evaluated by using Fisher’s exact test. A *p*-value < 0.05 was considered statistically significant. All analyses were performed using R version 2.15.0 c [27].

### Neoadjuvant chemoradiotherapy

All patients underwent a standardized neoadjuvant chemoradiotherapy (nCRT) treatment, including Taxol administration and 41.4 Gy irradiation. A prolonged interval between completion of the neoadjuvant treatment and surgery was chosen.

### Hybrid Ivor Lewis esophagectomy

Ivor Lewis esophagectomy has successfully been performed in our department for the treatment of esophageal cancer for the past 25 years [28, 29]. To address the issue of pulmonary complications, representing a frequently occurring morbidity associated with open surgery [29, 30], and to reduce postoperative pain, we adopted a robotic approach to the thoracic phase of this operation in 2015. On the other hand, to obtain optimal gastric tube mobilization and preparation we have continued to perform the abdominal phase of the Ivor Lewis esophagectomy by open surgery. The abdominal part of the surgery for abdominal lymph node dissection and construction of the gastric conduit was performed in the same manner in the COSU and the LIST cohort. The gastric conduit was created by using multiple Endo GIA™ cartridges. Initially, one Endo GIA™ 45 mm stapler with a black cartridge was set horizontally slightly proximal of the angular incisure. Next, multiple Endo GIA™ 60 mm with black cartridges were placed toward cranial to create the gastric tube with a width of approximately 4 cm. The staple line was then further secured with multiple stitches with Monocryl. After completion of the abdominal part, the patient was placed in a left-semi prone position and isolated left lung ventilation was achieved by using a double lumen endotracheal tube. A mini- thoracotomy was performed, four robotic ports inserted, and the robotic arms were docked. During the thoracic phase, mediastinal lymph node dissection and the intrathoracic anastomosis were performed. This hybrid Ivor Lewis esophagectomy including an open abdominal and a robotic thoracic phase was used throughout the present study.

All surgeries were performed by the same highly experienced senior surgeon who has performed over 500 robotic surgeries.

### Gastroesophageal anastomosis

**Fig. 1:**
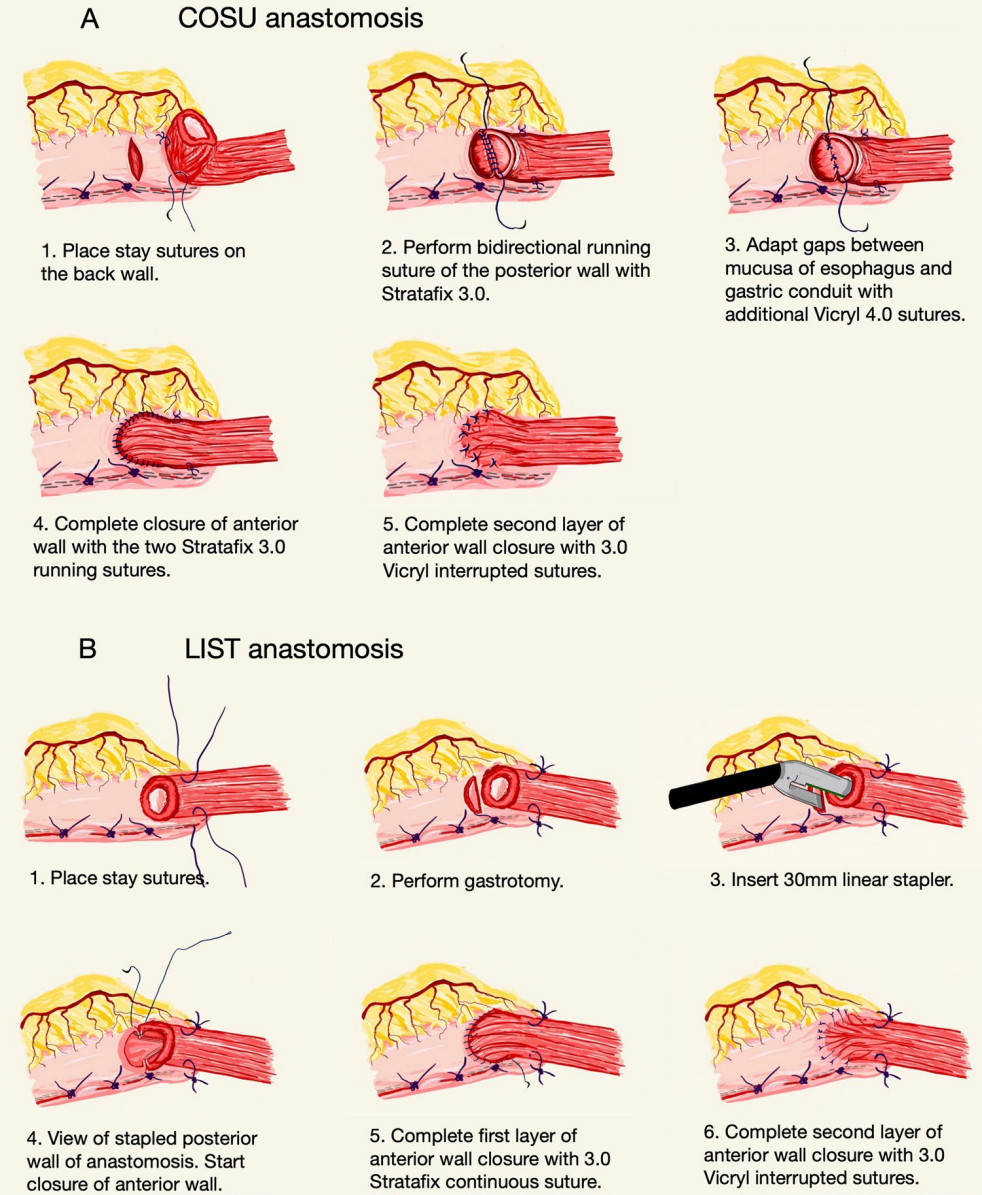
Different steps of COSU (**A**) gastroesophageal sutures. Different steps of LIST (**B**) gastroesophageal sutures

Based on previous reports [7], in the initial phase of our study, we created gastroesophageal anastomoses by LIST technique in 21 patients (see below) (Fig. 1A). In particular, the posterior wall of the anastomosis was adapted using the 30 mm daVinci Stapler with a green cartridge [31], while the anterior wall was sutured with two Stratafix™ 3.0 continuous sutures and an additional layer of single 3.0 Vicryl® stitches.

However, a thorough internal data review and further review of the literature suggested that the LIST technique might affect perfusion of the anastomosis by limiting blood supply to the blind end of the gastric conduit on the opposite side of the gastroepiploic artery [7, 32]. Moreover, also considering that both nCRT and stapling had previously been suggested to be associated with subsequent development of anastomotic strictures [33, 34], in the following 32 patients, we performed the gastroesophageal anastomoses by COSU.

The end-to-side esophagogastrostomy by COSU technique was performed using a continuous Stratafix™ 3.0 bidirectional spiral knotless suture (Fig. 1B). Before starting the running suture from the posterior wall toward both sides of the anterior part, two Vicryl® 3.0 stay sutures were placed to hold the lumen wide open. With this support, the stiches could be placed under a better view with full thickness technique. If small gaps between the mucosa of the esophagus and the conduit occurred while suturing the back layer of the anastomosis, additional Vicryl® 4.0 single stiches were carefully placed for further adaptation without compromising perfusion. After completion of the circular anastomosis, the two ends of the continuous sutures were knotted and the anastomosis was secured with a second layer of 3.0 Vicryl® interrupted sutures.

In either case, indocyanine green was used to assess the perfusion of the tissue. Furthermore, an omental patch was placed over the anastomosis and attached to the pleura with three Vicryl® 3.0 sutures in both techniques. After irrigation, two rigid chest tubes were placed in the thoracic cavity with one tip in an apical and the other in a basal position.

A video of the COSU and LIST techniques utilized throughout this study is enclosed.

### Postoperative regimen

All patients underwent the same postoperative regimen. In particular, all of them were admitted to the intensive care unit and as soon as no further ventilation or circulatory support was needed patients were transferred to the intermediate care unit, warranting further meticulous monitoring. Total parenteral nutrition was initiated. The nasogastric tube was left in situ for 7 days. On the 7th postoperative day, a radiological control of the anastomosis was performed and the nasogastric tube was removed. Patients were transferred to the general surgical ward according to their clinical course and peroral liquids started to be administered. Regular diet was then progressively resumed.

## Results

### Patients

Between 2015 and 2020, 53 patients with esophageal cancer were treated in a single institution with a standardized nCRT protocol before undergoing hybrid robot-assisted Ivor Lewis esophagectomy. Anastomosis was performed by COSU in 32 patients and LIST in 21 patients (see above). The two groups were similar, regarding patients’ age, sex and ASA, histological tumor type, tumor location and TNM or UICC tumor stage (Table 1).

**Table 1:** Demographic and clinical data of patients treated by robot-assisted Ivor Lewis esophageal surgery with continuously sutured (COSU) or linear-stapled (LIST) anastomosis

	COSU *n* = 32	LIST *n* = 21	*p*
Age: median (IQR)	68.5 (65.5–72)	70 (67–75)	0.647
Female: *n* (%)	6 (18.7%)	5 (23.8)	0.735
ASA score			
II: *n* (%)	13 (40.6)	7 (33)	
III: *n* (%)	18 (56.2)	13 (61.9)	
IV: *n* (%)	1 (3.1)	1 (4.7)	0.844
Histology			
Adenocarcinoma: *n* (%)	28 (87.5)	20 (95.2)	
Squamous cell carcinoma: n (%)	3 (9.3)	1 (4.7)	
Neuroendocrine tumor: *n* (%)	1 (3.1)	0	0.794
Location			
Middle esophagus: *n* (%)	3 (9.3)	1 (4.7)	
Distal esophagus: *n* (%)	12 (37.5)	8 (38)	
Siewert 1: *n* (%)	9 (28.1)	3 (14.2)	
Siewert 2: *n* (%)	8 (25)	8 (38)	
Siewert 3: n (%)	0	1 (4.7)	0.682
TNM stage			
cT2: *n* (%)	6 (18.7)	3 (14.2)	
cT3: *n* (%)	25 (78.1)	17 (80.9)	
cT4: *n* (%)	1 (3.1)	1 (4.7)	0.881
cN0: *n* (%)	22 (68.7)	17 (80.9)	
cN + : *n* (%)	10 (31.2)	4 (19)	0.362
cM0: *n* (%)	32 (100)	21 (100)	
cM + : *n* (%)	0	0	1
UICC stage			
1: *n* (%)	5 (15.6)	0	
2: *n* (%)	7 (21.8)	7 (33)	
3: *n* (%)	20 (62.5)	14 (66)	0.376

### Neoadjuvant chemoradiotherapy

The neoadjuvant treatment was similarly effective in both groups of patients, with a median Mandard Tumor Regression Grade [35] of 3 (IQR 2–4 and 2–3 for the COSU or LIST cohorts, respectively). PET studies, performed in a subset of patients, confirmed tumor regression upon nCRT in both groups with PET-SUV values decreasing from 10.9 to 7.3 (*n* = 6, median:8, IQR 6.22–15.85,) to 3.4–1.99 (median 2.35, IQR 2.12–4.9) (p = 0.02) in the COSU cohort, and from 13.12 to 8.2 (*n* = 19, median 12, IQR 7.1–17.6) to 3.6–1.65 (median 3.8, IQR 3.1–4.4) (*p* = 0.009) in the LIST cohort. Surgery was performed 73–17 days after nCRT termination in the COSU cohort (median 70, IQR 63–76) and 72–11 days after in the LIST cohort (median 69, IQR 64–79) (*p* = 0.788).

### Perioperative data and surgical outcomes

These results are listed in Table 2. The duration of operation was similar in patients with COSU or LIST anastomosis (median: 467 vs. 453 min, IQR 420–521 vs. 416–469).

**Table 2:** Perioperative results in patients undergoing robot-assisted Ivor Lewis esophagectomy with continuously sutured (COSU) or linear-stapled (LIST) anastomosis

	COSU *n* = 32	LIST *n* = 21	*p*
Operation time: median minutes (IQR)	467 (420–521)	453 (416–469)	0.0611
Morbidities			
None: *n* (%)	9 (28.1)	4 (19)	0.528
Minor: *n* (%)	19 (59.3)	13 (61.9)	1
Major: *n* (%)	4 (12.5)	4 (19)	0.697
CCI*: median (IQR)	21.75 (0–33.5)	26.2 (20.9–45.6)	0.224
Anastomotic leakage: *n* (%)	3 (9.3)	2 (9.5)	1.0
Pleural empyema: *n* (%)	1 (3.1)**	2 (9.5)	0.555
ICU stay: median days (IQR)	6 (5–7.25)	7 (6–11)	0.255
Hospital stay: median days (IQR)	20 (17–22)	21 (18–28)	0.039***
Mortality: *n* (%)	1 (3.1)	1 (4.7)	1

*Comprehensive complication index

**Observed in a case with anastomotic leakage

***Significant values

Similar numbers of patients experienced postoperative morbidities. In particular, minor complications were observed in 19/32 (59.3%) and 13/21 (61.9%) patients with COSU or LIST anastomosis, respectively (*p* = 1.0). These included pneumonia in 4/32 (12.5%) and 6/21 (28.5%) patients that received a COSU or LIST anastomosis, respectively (*p* = 0.168). Pneumothorax occurred in 2/32 (6.2%) and 1/21 (4.7%) patients (*p* = 1.000).

Major complications occurred in 4/32 (12.5%) patients with COSU and in 4/21 (19%) patients with LIST anastomosis (*p* = 0.697). Anastomotic leakage requiring treatment was observed in 3/32 (9.3%) and 2/21 (9.5%) (*p* = 1.0) patients with COSU or LIST anastomosis, respectively. Pleural empyema occurred in 1/32 (3.1%) and 2/21 (9.5%) of cases, respectively (*p* = 0.555).

Of the three patients with COSU anastomosis and anastomotic leakage, the leakage was treated with stent application and antibiotics in two cases. In one of these, treatment included the use of an Endo-Sponge®. In a third case, a stent was also used, but the patient experienced mediastinitis and pleural empyema requiring drainage. The patient later developed severe arrythmia requiring electrical conversion and deceased due to hemodynamical instability caused by the severe arrythmia. In a fourth patient a delayed wound healing occurred in the anastomotic area, but no leakage was observed.

In patients with LIST anastomosis, leakage occurred in two cases. One of them additionally developed a mediastinitis and a Pseudomonas aeruginosa sepsis and was treated with a stent and antibiotics. The second case was further complicated by pleural empyema and was initially treated with stent application as well as video assisted thoracoscopic surgery (VATS). Necrosis of the conduit required the resection of the anastomosis. The patient later developed a severe sepsis and deceased due to multiple organ dysfunction. Pleural empyema also occurred in a further case, necessitating a mini thoracotomy for effusion removal. In another patient, aspiration pneumonia required reintubation.

The Comprehensive Complication Index (CCI) did not differ with a median of 21.75 (0–33.5) in COSU and 26.2 (20.9–45.6) in LIST (*p* = 0.224).

In-hospital mortality was similar in the two groups of patients (1/32, 3.1% in COSU and 1/21, 4.8% in LSIT, *p* = 1.0).

Median intensive care unit (ICU) stay did not differ in patients after COSU or LIST anastomosis (*p* = 0.255), whereas a slightly, but significantly (*p* = 0.039) shorter overall hospital stay was observed for patients with COSU, as compared to LIST anastomosis with a median of 20 (17–22) in COSU and 21 (18–28) in LIST (*p* = 0.039).

The 90-day follow-up revealed no significant differences between the COSU and the LIST cohort. These results are shown in Table 3. In COSU rehospitalization occurred in 2/32 (6.3%). This was due to one case of dysphagia and in another case due to dyspnea. In the latter patient, further diagnostics revealed pleural effusion due to a recurrence with pleural carcinomatosis. An endoscopy was performed in 7/32 (21.9%)—in four cases due to dysphagia, in one case due to indigestion, in another case due to food bolus impaction and in the last case it was a planned endoscopic control of the anastomosis. Anastomotic stenosis was observed in 1/32 (3.1%) patients and was treated with balloon dilation. In the LIST cohort rehospitalization occurred in 3/21 (14.3%) patients. In one patient this was due to arrythmia, in another due to emesis caused by a viral infection and in the last case it was a planned rehospitalization for an endoscopic control and stent removal. Endoscopy was performed in 3/21 (14.3%) patients—in the latter two rehospitalized patients and in a third case due to dysphagia. Stenosis or local recurrence were not seen in any patient in the LIST cohort. Following the index hospitalization, no further mortality was observed in either cohort. Thus, the 90-day mortality is equal to the in-hospital mortality.

**Table 3:** 90-day follow-up of patients that underwent robot-assisted Ivor Lewis esophagectomy with continuously sutured (COSU) or linear-stapled (LIST) anastomosis

	COSU *n* = 32	LIST *n* = 21	*p*
Rehospitalization: *n* (%)	2/32 (6.3)	3/21 (14.3)	0.374
Endoscopy *n* (%)	7/32 (21.9)	3/21 (14.3)	0.722
Stenosis: *n* (%)	1/32 (3.1)	0/21 (0)	1.000
Recurrence: *n* (%)	1/32 (3.1)	0/21 (0)	1.000
Mortality: *n* (%)	1/32 (3.1)	1/21 (4.8)	1.000

### Clinical–pathological data

A R0 resection was obtained in 30/32 (93.7%) patients with COSU and in all patients with LIST anastomosis (*p* = 1.000) (Table 4). Pathological staging of specimens from patients treated with robot-assisted Ivor Lewis surgery and COSU or LIST anastomosis was also similar.

**Table 4:** Clinical–pathological data from patients treated by robot-assisted Ivor Lewis esophagectomy with continuously sutured (COSU) or linear-stapled (LIST) anastomosis

	COSU *n* = 32	LIST *n* = 21	*p*
R0 resection: *n* (%)	30 (93.7)	21 (100)	
R1 resection: *n* (%)	2 (6.6)	0	1.0
ypT0: *n* (%)	6 (18.7)	5 (23.8	
ypT1: *n* (%)	6 (18.7)	2 (9.5)	
ypT2: *n* (%)	6 (18.7)	7 (33.3)	
ypT3: *n* (%)	13 (40.6)	7 (33.3)	
ypT4: *n* (%)	1 (3.1)	0	0.631

## Discussion

Esophageal cancer surgery is highly demanding. Within this context, the role of robotic technology in comparison to other minimally invasive procedures has not yet been conclusively established. Nevertheless, considering the increasing relevance of its clinical use, different aspects of robotic technology deserve careful investigation.

Gastroesophageal anastomoses can be created by different techniques. In particular COSU, CIST and LIST have widely been used. A meta-analysis, addressing advantages and disadvantages associated with the use of these techniques has recently been published [16]. However, there is a lack of comparative studies by the same team, particularly regarding COSU and LIST, as preferred techniques for the creation of the anastomosis.

Importantly, although nCRT is presently widely used for the treatment of esophageal cancer prior to surgery, published studies most frequently include substantial percentages of patients undergoing surgery without preliminary nCRT, or following application of different nCRT protocols. This could pose an important bias since neoadjuvant treatment while reducing tumor mass, also affects surrounding healthy tissues, potentially increasing the rate of complications [20, 21]. The median interval between completion of the nCRT and surgery of 70 days in COSU and 69 days in LIST allowed patients to recuperate after the neoadjuvant treatment. Furthermore, we aimed to achieve an improved rate of complete histological response due to the chosen interval, which is what the literature reports for prolonged time until surgery [36–38].

To provide more solid bases for a comparative evaluation, in this study we analyzed perioperative morbidities and clinical outcomes in two groups of patients with esophageal cancer operated in the same center with a hybrid robot-assisted Ivor Lewis procedure following a highly standardized nCRT, with anastomoses created by COSU or LIST technique.

Our data indicate that LIST is not superior to COSU in hybrid robotic esophagectomy following nCRT. A minor, non-significant decrease in operation time in surgeries with LIST anastomosis was indeed detected. However, number and severity of complications, which were overall in agreement with currently published data [39], were similar in patients operated with LIST or COSU anastomosis. Instead, a slightly, though significantly, shorter duration of overall hospital stay even emerged for patients with COSU anastomosis. This might be, at least in part, due to the different treatments required for anastomotic leakage and pleural empyema. Whereas a pleural empyema occurring in a patient with COSU anastomosis could be treated by drainage, in the two cases of pleural empyema observed in patients with LIST anastomosis, a mini thoracotomy and a VATS were necessary. Furthermore, while the cases of anastomotic leakages in the COSU cohort were treated with stents and Endo-Sponge®, one case of an anastomotic leakage in LIST required the resection of the anastomosis due to conduit necrosis. Although no significant differences were observed regarding the morbidity and the Comprehensive Complication Index, nevertheless the differences in the required treatments have likely had an impact the overall length of hospital stay.

Most importantly however, leakage was detectable in similarly small numbers of patients with COSU or LIST anastomosis.

Limitations of our study should be acknowledged. In particular, the number of treated patients is relatively small and they were not randomized. Furthermore, endoscopy was not routinely performed, and non-significant leakages may have been missed. However, nCRT standardization and the fact that all operations were performed by the same team of surgeons, reinforce the relevance of our data.

While larger, and possibly randomized studies will be needed to validate these results, they nevertheless support a non-inferiority of COSU, as compared to LIST anastomosis in patients undergoing robot-assisted Ivor Lewis esophagectomy following nCRT.

It is tempting to speculate that while LIST anastomosis clearly provides a major advantage in the suture of the posterior wall of gastroesophageal anastomosis during thoracoscopic esophagectomy, its superiority over COSU might fade in robot-assisted surgery, characterized by enlarged 3D view, longer and articulated instruments and reduction of physiologic tremor.

## Supplementary Information

Below is the link to the electronic supplementary material.

Supplementary file1 (MP4 250042 KB)

Supplementary file2 (TIF 2700 KB)
