# Supplementary figures and images for: Continuously sutured versus linear-stapled anastomosis in robot-assisted hybrid Ivor Lewis esophageal surgery following neoadjuvant chemoradiotherapy: a single-center cohort study
Source: Surg Endosc. 2022 Jul 19;36(12):9435–43. doi: 10.1007/s00464-022-09415-3 (PMC9652283; doi:10.1007/s00464-022-09415-3)

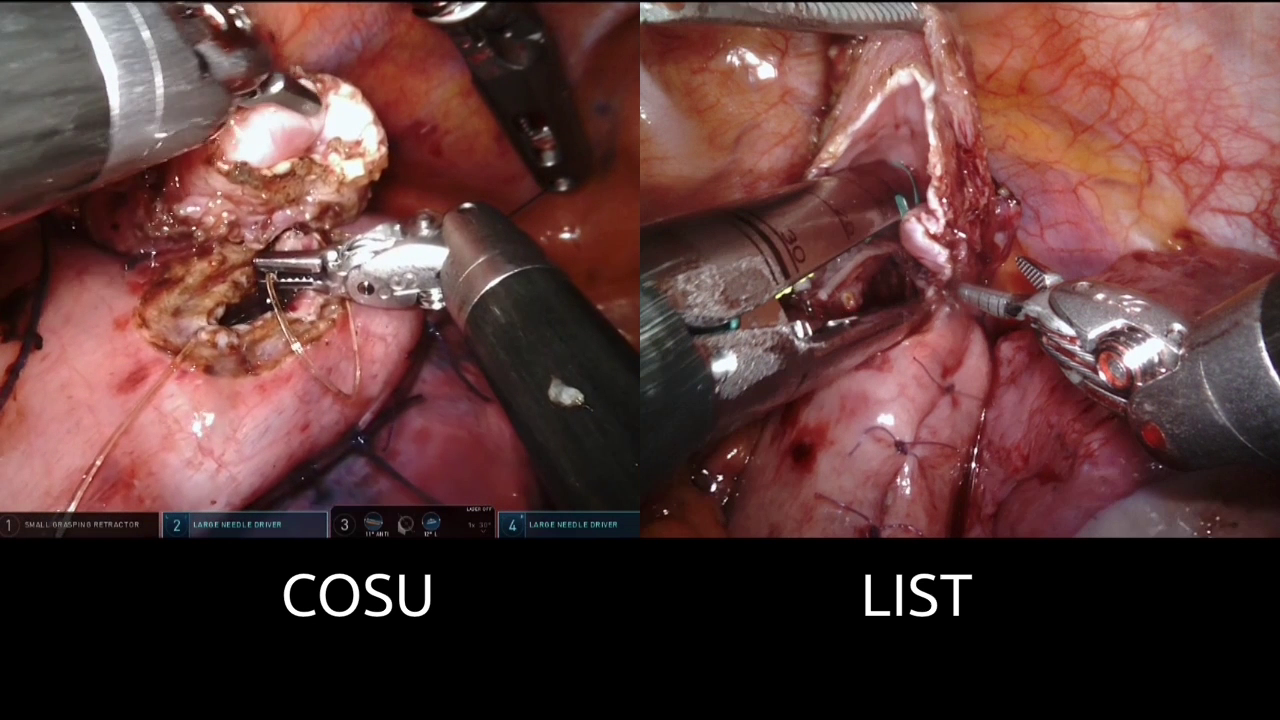

Supplement: Supplementary file 2 — Supplementary file2 (TIF 2700 KB) [file 464_2022_9415_MOESM2_ESM.tif]
